# Effect of HIV infection on the acute antibody response to malaria antigens in children: an observational study

**DOI:** 10.1186/1475-2875-10-55

**Published:** 2011-03-05

**Authors:** Daniel KM Muema, Francis M Ndungu, Samson M Kinyanjui, James A Berkley

**Affiliations:** 1 Centre for Geographic Medicine Research (Coast), Kenya Medical Research Institute, P.O. Box 230, Kilifi, 80108, Kenya; 2 Centre for Clinical Vaccinology & Tropical Medicine, University of Oxford, Oxford, UK

## Abstract

**Background:**

In sub-Saharan Africa, the distributions of malaria and HIV widely overlap. Among pregnant and non-pregnant adults, HIV affects susceptibility to malaria, its clinical course and impairs antibody responses to malaria antigens. However, the relationship between the two diseases in childhood, when most deaths from malaria occur, is less clear. It was previously reported that HIV is associated with admission to hospital in rural Kenya with severe malaria among children, except in infancy. HIV-infected children with severe malaria were older, had higher parasite density and increased mortality, raising a hypothesis that HIV interferes with naturally acquired immunity to malaria, hence with little effect at younger ages (a shorter history of exposure). To test this hypothesis, levels of anti-merozoite and schizont extract antibodies were compared between HIV-infected and uninfected children who participated in the original study.

**Methods:**

IgG responses to malaria antigens that are potential targets for immunity to malaria (AMA1, MSP2, MSP3 and schizont extract) were compared between 115 HIV-infected and 115 age-matched, HIV-uninfected children who presented with severe malaria. The children were classified as high and low responders for each antigen and assigned antibody-response breadth scores according to the number of antigens to which they were responsive. A predictive logistic regression model was used to test if HIV was an effect modifier on the age-related acquisition of antibody responses, with age as a continuous variable.

**Results:**

Point estimates of the responses to all antigens were lower amongst HIV-infected children, but this was only statistically significant for AMA1 (P = 0.028). HIV-infected children were less likely to be high responders to AMA1 [OR 0.44 (95%CI, 0.2-0.90) P = 0.024]. HIV was associated with a reduced breadth of responses to individual merozoite antigens (P = 0.02). HIV strongly modified the acquisition of antibodies against schizont extract with increasing age (P < 0.0001), but did not modify the rate of age-related acquisition of responses to individual merozoite antigens.

**Conclusions:**

In children with severe malaria, HIV infection is associated with a lower magnitude and narrower breadth of IgG responses to merozoite antigens and stunting of age-related acquisition of the IgG antibody response to schizont extract.

## Background

HIV and malaria are major causes of morbidity and mortality in sub-Saharan Africa [1]. Within the region, there is widespread overlap in the distribution of the two diseases [2]. Thus, any interaction between the two diseases may have potentially important public health implications.

There is evidence that HIV infection influences susceptibility to, and the clinical course of malaria. Studies in non-pregnant [3-7] and pregnant adults [8-10] suggest that HIV infection is associated with more frequent episodes of clinical malaria and higher parasite density. However, reports of the effects of HIV on malaria in childhood, when most malaria deaths occur, have been inconsistent.

Naturally acquired immunity to malaria is dependent on exposure. Thus, in malaria endemic areas, immunity to severe disease, mild disease and parasitaemia normally increases with age [11,12]. A recent report from Kilifi, Kenya suggested that HIV infection is associated with hospital admission for severe malaria among children [13]. Importantly, those infected with HIV were older (median age, 38 months; IQR, 26-63 months) than those without HIV infection (median age, 19 months; IQR, 10-35 months; P < 0.001). HIV-infected children had higher peripheral parasite density when corrected for age. Despite the overall strong association between HIV infection and severe malaria, there was no relationship between HIV and severe malaria in infancy [13]. This raised the hypothesis that HIV might stunt the age-related acquisition of natural immunity to malaria, thus having little effect among the youngest children who have not yet acquired natural immunity to malaria.

Since both the breadth and magnitude of IgG antibody responses to multiple *Plasmodium falciparum *merozoite antigens have been associated with immunity to clinical malaria [14], this study was conducted to investigate the effects of HIV infection on the antibody response to three merozoite antigens that are potential targets for immunity to malaria: apical merozoite antigen 1 (AMA1), merozoite surface protein 2 (MSP2) and merozoite surface protein 3 (MSP3); and whole parasite schizont extract.

## Methods

### Location and study population

Kilifi District Hospital, Kenya, serves approximately 240,000 people in a rural, coastal area where malaria is endemic (<1 to 120 mosquito bites are infective for *P. falciparum *each year) [15]. This nested study was conducted using plasma samples collected during a prospective study undertaken between 1998 and 2002 [13]. The prevalence of HIV infection was 1.7% among children sampled in the community during the study period, and 9.8% among women attending the hospital antenatal clinic in 2000[13]. At the time of the study there were no specific HIV care services and consequently no use of anti-retroviral drugs or co-trimoxazole prophylaxis. This 'natural history' study would not now be possible, because of the current widespread use of these drugs. Approval for the study was given by the Kenyan National Ethical Review Committee (SSC No. 502 and SCC No. 485) and individual written informed consent was obtained.

### Clinical data collection

Trained research clinicians provided care and collected standardized clinical and laboratory data on all paediatric admissions. Clinical features of severe malaria were defined as either a history of febrile illness or an axillary temperature ≥37.5°C on admission, plus one or more of the following signs: impaired consciousness (the inability to localize a painful stimulus if aged >8 months, or not having directed eye movements if age ≤8 months), deep breathing (Kussmaul's respiration) or severe anaemia (haemoglobin <5 g/dl) [13]. Children received supportive care and anti-malarial treatment according to national guidelines and WHO recommendations.

Malaria diagnosis was performed by examining Giemsa-stained thick and thin blood-smears at x1,000 magnification. A full blood count was performed using an MD2 automated counter (Beckman/Coulter, UK). Peripheral parasite densities were calculated from each child's actual red and white cell counts. HIV testing was done retrospectively by enzyme-linked immunosorbent assay after delinking the samples from their identity data; true infection was confirmed by polymerase chain reaction for children aged <18 months (Ampliclor; Roche). HIV infection was present in 133 (12%) of the 1,071 parasitaemic children with signs of severe malaria[13].

### Recombinant antigens

The recombinant MSP2 CH150/9 was expressed in *Escherichia coli *[16]. MSP3 (full length) was expressed in *Escherichia coli *[17]. Recombinant AMA1 (1:1 mixture of FVO and 3d7) was expressed in *Pichia pastoris *[18].

### Antibody assays

The study was conducted using stored plasma samples taken at the time of admission with a diagnosis of severe malaria from 115 HIV-infected children and 115 samples randomly drawn from the larger set of HIV-uninfected children matched on age and season (wet/dry) of admission to eliminate their potential confounding effects.

Enzyme-linked immunosorbent assays (ELISAs) on antibodies against parasite schizont extract and recombinant antigens AMA1, MSP2 and MSP3 were performed as previously described [14]. Briefly, individual wells of microtitre plates (Nunc) were coated with 50 ng of antigen in 100 μl of carbonate coating buffer (15 mM Na2CO3, 35 mM NaHCO3, pH 9.3). Alternatively, microtitre plates were coated with *P. falciparum *schizont extract (the A4 strain) in phosphate-buffered saline (PBS) according to the method of Ndungu *et al *[19]. Plates were incubated overnight at 4°C before washing three times in PBS-Tween (PBS-0.05% Tween 20) and subsequently blocking for five hours at room temperature with 1% skimmed milk in PBS-Tween. Following this, plates were washed four times and thereafter incubated overnight at 4°C with 100 μl of test serum (1/500 dilution in blocking buffer). Plates were then washed four times and then incubated for one hour at room temperature with 100 μl of alkaline phosphatase conjugated goat antihuman IgG (Sigma) at 1/500 in blocking buffer. They were then washed four times and subsequently incubated for 20 minutes at room temperature with 50 μl of diluted p-nitrophenyl phosphate (Sigma) for color development. The reaction was stopped using 50 μl of 3 M NaOH. Optical densities were measured at 405/570 nanometers.

A single adult hyper-immune serum, with an assigned IgG antibody concentration in Arbitrary Units (AU), was serially diluted and included in each plate. It was later used to convert the optical density (OD) readings of the samples into relative concentrations in AU, thereby eliminating the effect of inter-plate and inter-day variation.

### Statistical analysis

The data were analysed with STATA version 11.0 (Stata Corporation, TX, USA). P values < 0.05 were considered as significant. The Chi squared test was used to compare proportions. As antibody levels were skewed, the Wilcoxon signed-ranks test for matched pairs was used to compare antibody levels (AU) between children with and without HIV infection. Each child was categorized as a high or low responder to each antigen depending on whether their antibody levels were above or below the overall median. Conditional logistic regression, adjusted for age, season, parasite density, haemoglobin and residence in the Northern or Southern part of the district (the two areas have different malaria transmission intensities[20]) was used to determine the odds of a child being a high responder to each antigen. Breadth of merozoite responses was assessed by assigning the children a score on the basis of the number of antigens to which they were high responders i.e. zero to three (response to schizont extract was not included in the score). The Chi squared test for trend was used to test the relationship between breadth of response and HIV status.

To test if HIV infection was acting as an effect modifier on the age-related acquisition of antibody response, a predictive logistic regression model was used with the probability of being a high responder as the dependent variable and age (continuous) and HIV status as independent variables according to the method of Garrett [21] and adjusted for season, parasite density, haemoglobin and residence in the Northern or Southern area. This model estimated the probability of a child being a high responder as age increased, stratified by HIV status. The P value of the likelihood ratio test (LRT) for the interaction of age*HIV status is reported.

## Results

### Characteristics of the study population

The median age of the HIV infected children included in this analysis was 37 months (interquartile range (IQR) of 25 to 59 months) and that of the age-matched HIV uninfected children was 37 months (IQR 25 to 59 months). Two HIV-uninfected and four HIV-infected children had concurrent bacteraemia. Eight HIV-uninfected and fourteen HIV-infected children died. There were no significant differences between the two groups with regard to nutritional status and the three main clinical features of severe malaria (i.e. anaemia, unconsciousness and deep breathing) (Table 1).

**Table 1:** Population characteristics

	HIV-uninfected	HIV-infected	P value
N	115	115	
Age in months median (Interquartile range)*	37 (25-59)	37 (25-59)	1.00
Parasite density median (Interquartile range)	26441 (2860-124800)	40413 (3039-148780)	0.90
Severely anaemic^1^	68	68	0.87
Unconscious^2^	39	45	0.47
Bacteraemia^3^	2	4	0.42
Deep acidotic breathing	27	24	0.58
Malnutrition^4^	30	34	0.61
Death	8	14	0.19

* Groups were matched on age

^1^haemoglobin < 5 g/dl

^2 ^inability to localize a painful stimulus for children aged 18 months or lack of directed eye movements for infants aged <8 months

^3 ^positive aerobic blood culture for pathogenic bacteria

^4 ^severe underweight (weight-for-age z score, <3) or having kwashiorkor

### Antibodies to merozoite antigens and HIV infection status

**Figure 1:**
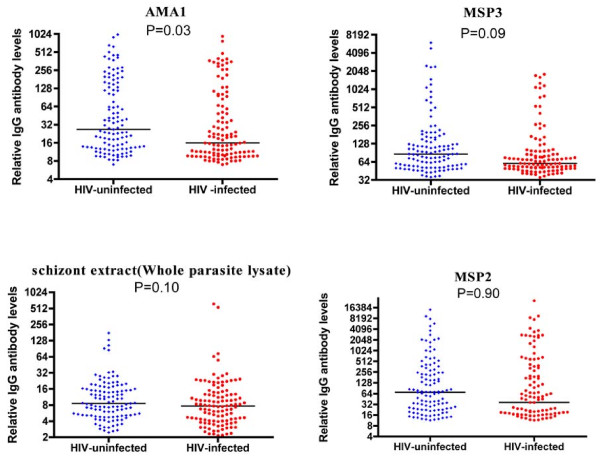
**Relative levels of antibody to malaria antigens among HIV-infected and HIV-uninfected children**.

**Figure 2:**
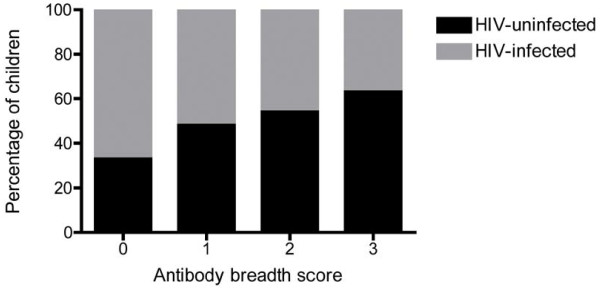
**Breadth of response scores for three merozoite antigens among HIV-infected and HIV-uninfected children**. Chi squared test for trend P = 0.02

The point estimates of levels of antibodies to all the antigens tested were lower among HIV-infected children. This was statistically significant for antibodies against AMA1 (P = 0.028) but not for those against MSP2 (P = 0.90), MSP3 (P = 0.09) or schizont extract (P = 0.10) (Figure 1). HIV infected children were less likely to be high responders to AMA1 [OR 0.44 (95%CI, 0.2-0.90) P = 0.024]. There was no significant difference with regard to MSP2 [OR 0.68 (95%CI, 0.31-1.46) P = 0.32], MSP3 [OR 0.74 (95% CI, 0.34-1.59) P = 0.44] or schizont extract [OR 0.67 (95% CI 0.36 - 1.57) P = 0.36]. HIV infection interfered with the breadth of antibody response to merozoite antigens. The proportion of HIV infected children decreased as breadth score increased (Chi squared test for trend P = 0.02, Figure 2).

**Figure 3:**
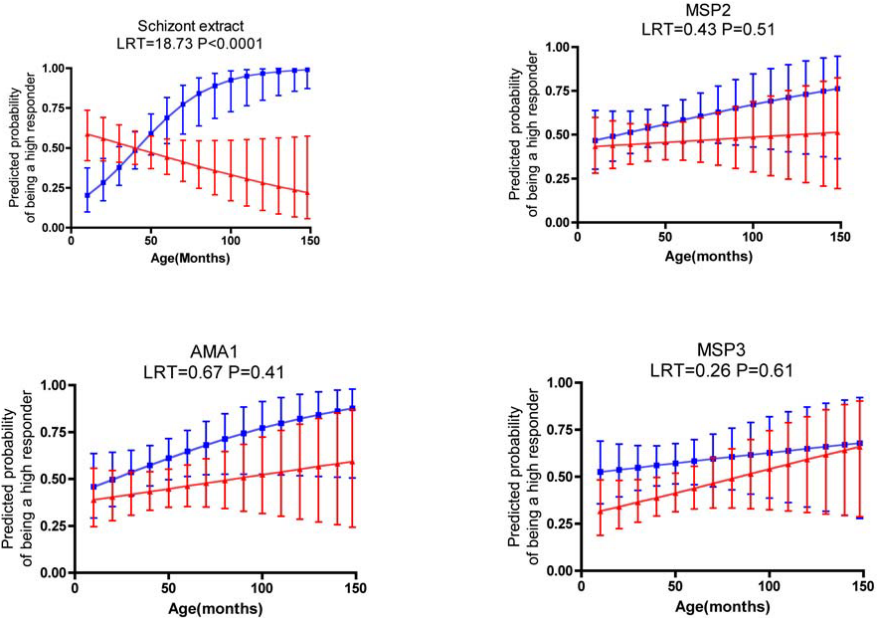
**Probability of being a high responder against the various antigens with increasing age**. Blue represents HIV-uninfected children while red represents HIV-infected children. Error bars indicate the 95% confidence intervals. LRT refers to the likelihood ratio test for the interaction of age*HIV status in the logistic regression model

HIV infection was a strong effect modifier on the age-related acquisition of antibody response against the schizont extract (LRT = 18.73, P < 0.0001), with no increase in the probability of being a high responder against schizont extract with increasing age among HIV-infected children (Figure 3). However, there was no evidence for effect modification by HIV on the age-related acquisition of responses to the individual merozoite antigens: AMA1 (LRT = 0.67, P = 0.41), MSP2 (LRT = 0.43, P = 0.51) and MSP3 (LRT = 0.26, P = 0.61). Thus, although the point estimates of the probabilities of being a high responder were consistently lower among the HIV-infected children, estimates of the probability of being a high responder for these individual merozoite antigens increased with age, regardless of the HIV status (Figure 3).

## Discussion

This study suggests that HIV-infected children have impaired ability to respond to multiple malaria antigens and HIV modifies the age-related acquisition of antibodies to *P. falciparum *whole parasite schizont extract. Thus, HIV infection impairs both the magnitude and breadth of antibody response. Since antibodies are important in immunity to severe malaria [22], these results are compatible with the previous epidemiological observations which suggested that HIV stunts the age-related acquisition of immunity to severe malaria (increased age, parasite density and case fatality) [13]. The inconsistent findings of studies of the association between HIV and malaria in children may therefore reflect the fact that the observed effect of HIV on malaria is likely to depend on both age and transmission intensity.

The response to schizont extract is a reflection of the total response to the many individual antigens in the malaria parasite. As such, the observed effect modification by HIV on the age-related acquisition of response is likely to reflect the cumulative effect modification on the acquisition of responses to many malaria antigens, which might not be observed if the individual responses are studied separately.

The stunting of age-related acquisition of antibodies to malaria antigens by HIV observed in this study parallels the impaired parity-dependent acquisition of antibodies against the variant surface antigens associated with placental malaria parasites reported among Malawian HIV-infected pregnant women [23]. Consistent with the results of this study the HIV-infected women also had reduced antibodies against AMA1. Similarly, among non-pregnant Zambian adults, HIV impaired the antibody response to AMA1 but spared antibody responses to MSP2 [24].

Previous studies among adults have reported that the risk of malaria infection and severe disease rises with increasing derangement of the immune system due to HIV and is highest among individuals with the lowest CD4 cells counts [3,7,25-27], suggesting that the interaction between HIV and malaria is immunological. However, the mechanism by which HIV affects antibody responses to malaria is unclear. In more advanced stages of HIV infection, malaria responses are probably stunted along with the global stunting of all immune responses. One possible specific mechanism by which HIV could stunt IgG responses to parasites, including malaria, is through the HIV Nef, an HIV viral protein, which accumulates in B cells and prevents class switching of HIV and non-HIV responses [28,29]. However, observations in this study and the other studies cited above suggest that in the earlier stages of infection, HIV also influences specific malaria responses.

It can be argued that, in one sense, protection against severe malaria had failed in all of these children since they all had severe malaria. However, responses differed between HIV-infected and uninfected children, suggesting true differences in rapid antibody responses during this severe episode. Furthermore, in the original study, the HIV infected children had higher parasitaemia and were more likely to die, suggesting that they were more immunologically deficient in terms of their ability to control the malaria episode.

The study was limited to three malaria merozoite antigens and the whole parasite lysate. The effect of HIV on the variant surface antigens (VSAs) and the remaining thousands of malaria antigens, the majority of which are yet to be expressed and purified into recombinant proteins, remains unexplored. Additionally, the sample size was restricted to the number of available HIV-infected plasma samples from the parent study. There were no data on CD4 counts or HIV viral loads from the children in this study.

## Conclusions

In children with severe malaria, HIV infection is associated with a lower magnitude and narrower breadth of IgG responses to merozoite antigens and stunting of age-related acquisition of antibody responses to schizont extract. Further research should investigate fractionated schizont extract to identify the most significant components; the effect of HIV on avidity maturation; functionality of the antibodies; and the ability of HIV-infected children to mount effective responses to current and putative malaria vaccines.

## Conflict of interests

The authors declare that they have no competing interests.

## Authors' contributions

JAB and SMK conceived the study. JAB undertook patient care and sample collection. Laboratory methods were designed by DKMM, FMN and SMK and undertaken by DKMM and FMN. Analysis was conducted by DKMM and JAB. The manuscript was drafted by DKMM and critically reviewed and edited by all authors.
